# Comprehensive comparison of treatments for controlling the large pine weevil (*Hylobius abietis*) in Central Europe

**DOI:** 10.1038/s41598-022-13729-6

**Published:** 2022-06-11

**Authors:** Juraj Galko, Michal Lalík, Slavomír Rell, Christo Nikolov, Marek Barta, Ján Pittner, Silvia Hyblerová, Milan Zúbrik, Andrej Kunca, Jozef Vakula, Andrej Gubka, Jaroslav Holuša

**Affiliations:** 1 National Forest Centre, Forest Research Institute Zvolen, Zvolen, Slovakia; 2 Faculty of Forestry and Wood Sciences, Czech University of Life Sciences Prague, Prague, Czech Republic; 3 Institute of Forest Ecology, Slovak Academy of Sciences, Nitra, Slovakia; 4 Faculty of Forestry, Technical University Zvolen, Zvolen, Slovakia; 5 Research Station of TANAP, Tatranská Lomnica, Slovakia

**Keywords:** Agroecology, Forestry

## Abstract

Adults of the large pine weevil (*Hylobius abietis*) cause serious damage to coniferous seedlings and are among the most important forest pests in Europe. Seedling protection by chemicals is gradually being restricted or banned for environmental reasons, and non-chemical alternatives are therefore needed. In this 3-year study, we compared the following five treatments for protecting Norway spruce seedlings against *H. abietis* in the Central European mountains where the weevil is especially abundant: alpha-cypermethrin sprays (the only chemical treatment); coating with sprayed glue (Vermifix); wax coating with C and F types (Norsk Wax); and physical protection with collars. The same block design was set up at a clear-cut site and at a nursery site to compare seedling mortality and wax quality under “wild conditions” with pests and under “ideal conditions” without pests. Repeated application of alpha-cypermethrin was the most effective and least expensive method to protect seedlings against *H. abietis*. Among the four non-chemical methods, repeated application of glue was the most effective. Because collars were moderately effective but not cost-effective, we do not recommend the use of collars. Wax was inexpensive and environmentally safe but protected seedlings for only 1 year; the newer F type of wax performed better than the C type of wax, and perhaps the F type can be improved. In general we found that seedlings at sites with high numbers of *H. abietis* require protection for at least 3 years. We conclude by providing an overview of all methods currently available for managing *H. abietis* in forests.

## Introduction

In large parts of Europe, successful regeneration of coniferous forests by planting depends on the suppression of damage caused by the large pine weevil, *Hylobius abietis* (Linnaeus, 1758) (Coleoptera: Curculionidae)^1–3^. This pest is especially damaging in young forest stands^2,4^.

*Hylobius abietis* adults are attracted to the fresh clear-cut by the odours emitted from damaged, dying or newly dead (cut) conifer trees providing the breeding substrate^5^; they remain at the site through the entire season and also overwinter at the site^6,7^. The individuals of the parent generation can survive several years and continue to lay eggs in the years following harvest. The new generation of weevils then emerge as adult weevils several years (1–3) later after the initial tree cutting^8^. Feeding of parental beetles to get reproductively mature when arriving to the clear-cut and losing their flight muscles. Feeding on the mature trees does not cause any economic damage, but beetle’s feeding on planted seedlings causes severe economic damage^9^, because feeding may cause girdling and often the death of coniferous seedlings^7,10,11^. A single weevil can damage several seedlings^12^. Larvae hatch soon after oviposition and develop under the bark of the above-mentioned material^13^. Development requires 1 to 3 years depending on the conditions^10,14^, but recent studies indicate that climate change (mainly increasing temperatures) may increase the feeding rate and shorten the weevil’s life cycle^15–17^. Maturing weevils also feed on the bark of coniferous seedlings^18,19^. *H. abietis* adults cause high levels of seedling mortality and economic losses^20^. In Europe, *H. abietis* causes annual damage of almost 120 million EUR^21^.

Depending on population density and weather conditions, *H. abietis* can kill 50–100% of seedlings at a site^11,22,23^. Current management relies on silviculture, feeding barriers, and insecticides^20^.

The use of synthetic insecticides is effective and relatively cheap^24^. However, because of adverse environmental effects, insecticide use will be increasingly restricted^2,19,24–26^ and replaced by various physical feeding barriers^27^. Of the 407 million conifer seedlings delivered in Sweden in 2020, for example, 50% were protected with stem coatings, 47% were unprotected, and only 3% were protected with insecticide^28^.

Physical barrier systems (glue/sand/wax coatings, paper/plastic guards, shields, collars, clipstops, etc.) are alternatives to synthetic insecticides. Substantial research on physical barriers has been conducted mainly in Scandinavian countries^3,27,29–31^, but also in the UK^11,24,32–34^, and Slovakia^19,21,37,38^.

A relatively new method for the physical protection of coniferous seedlings against the feeding damage caused by *H. abietis* is the application of Conniflex. In this method, the lower 60% of the seedling stem is protected with a coating containing fine sand embedded in an acrylate dispersion; application of Conniflex is an effective and environmentally friendly alternative to insecticide treatments^3,31^.

In Europe, wax (names during development: Bugwax, Eco-vax, KVAAE) has been used to protect seedlings against *H. abietis* for the last 10–15 years, although experiments and development have been ongoing since the 1990s^39^. This wax is made from natural materials. It does not contain any insecticide or fungicide and is harmless to insects and animals living in the forest. Wax is fully biodegradable, non-reactive, non-toxic, and insoluble in water^40^. The wax is significantly more elastic than normal wax, provides a physical barrier, reduces emission of volatile attractants, and thereby greatly reduces feeding by *H. abietis*^19,41^. In Slovakia, C type of wax (Norsk Wax) has been used to protect seedlings since 2013. The melted wax is manually applied to seedling stem or seedlings are inserted into a “fountain machine” that applies the wax; the wax is applied from the root collar to 15–20 cm above the root collar^19^. Under ideal conditions, the wax protects the seedlings for about 1.5–2.0 years. That this treatment can be as effective as insecticide application has been demonstrated in Sweden^27,30,42^ and Slovakia^19,21,37,38^.

Vermifix is a glue that was developed to protect trees against creeping insects and for use in various glue traps^43^. Vermifix (Papírna Moudry s.r.o., Czech Republic) has recently been tested for control of *H. abietis* on conifer seedlings^19,21,38^. In general, the coating of stems with glue or wax has been found to reduce *H. abietis* damage and in most cases provided control that was not significantly different from that provided by an insecticide treatment^21,38^.

Other potential alternatives are application of antifeedant compounds, the plant hormone methyl jasmonate (MJ), or natural product insecticides. The study of Azeem^44^ suggested that compounds produced by plants that are not hosts of *H. abietis* might be used to protect seedlings against *H. abietis* feeding. Unelius^45^ stated that research is needed to find compatible combinations of coating material and antifeedants that can protect seedlings against *H. abietis* feeding for two seasons without harming the seedlings. Recent research suggested that a MJ treatment may protect coniferous seedlings against insect herbivory^46,47^. Natural product insecticides are chemicals derived from plants or microorganisms. Willoughby^34^ described the effects of azadirachtin extracts from neem trees, pyrethrin extracts from the Dalmatian chrysanthemum, and even sheep fat on *H. abietis* feeding, but none of them led to protection of seedlings.

Clear-cutting followed by planting is the generally used method for the regeneration of coniferous forests in northern Europe^3,48^. In contrast, common Slovak forestry management is based on natural regeneration, i.e., a shelter-wood management system^49^ that normally results in a great number of naturally regenerated trees^50^. Under such conditions, pests such as *H. abietis* are not very significant. However, the annual occurrence of wind damage^51^, particularly in mountainous spruce regions, results in cleared areas that resemble areas subjected to clear-cut forest management. In these areas, the abundance of stumps and harvest residues provide breeding habitat and food for *H. abietis*^18^. Wind disturbances are also regularly followed by outbreaks of bark beetles (especially the spruce bark beetle, *Ips typographus*^52^), which produce additional material suitable for the development and maintenance of large populations of *H. abietis*. Damage to seedlings (mainly of spruce) by *H. abietis* is especially severe in national parks (at elevations up to 1200 m a.s.l., pers. observ.), where the use of insecticides for seedling protection is forbidden^21,38^. Therefore, researchers have recently investigated non-chemical alternatives for protecting coniferous seedlings against *H. abietis* feeding^18,19,21,37,38,53,54^.

**Figure 1:**
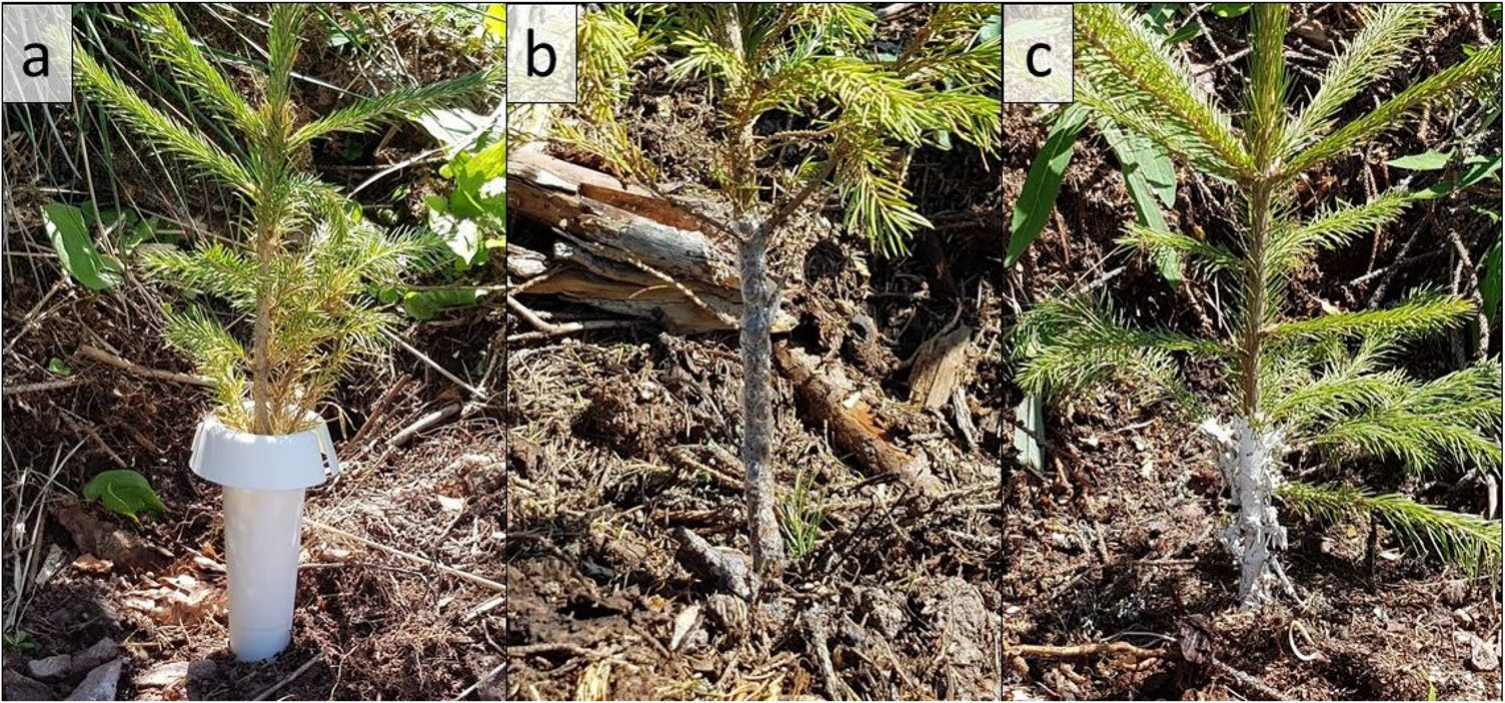
Spruce seedlings treated with (**a**) Hylopro, (**b**) Vermifix, and (**c**) Eco-wax.

In the current study, we conducted a 3-year experiment to compare various chemical (Fig. 1a–c) and non-chemical treatments for protecting seedlings against *H. abietis*. This experiment was conducted at a site in a mountainous region with high *H. abietis* abundance and at a nursery site where *H. abietis* was absent. We evaluated the efficacy, cost, and environmental effects of these treatments. Finally, we provide recommendations for *H. abietis* management under Central European conditions.

## Materials and methods

### Study sites

Experiments were performed at two sites. The first site was a forest nursery in Jochy (owner OZ Semenoles, LESY SR, state forest) (hereafter referred to as the nursery site), which is in the northern part of Central Slovakia in the Liptov region (49° 6′ 36.15″ N, 19° 44′ 58.36″ E, 835 m asl).

The second site was a clear-cut area located in the forest stand managed by the Forest district of Liptovská Teplička (OZ Liptovský Hrádok, LESY SR, state forest) (hereafter referred to as the clear-cut site) (48° 59′ 59.59″ N, 20° 2′ 14.07″ E, 1070–1100 m asl). The clear-cut site was a 3-ha area that had been recently cleared after sanitation logging of Norway spruce (*Picea abies* [L.] Karst.) (September 2017) due to *I. typographus* damage. The surrounding tree species were represented by spruce (95%) and larch (*Larix decidua* Mill.) (5%). The site contained approximately 230 spruce stumps per ha that had not been removed. Harvest residues has been placed in piles but had not been burned. The site is representative of the vast area of foothills of the Low Tatras Mts., which has a sufficient suitable habitat and food to support high numbers (25,000 weevils per ha) of *H. abietis*^54^.

### Seedlings, treatments, and experimental design

In spring 2018, 4-year-old Norway spruce seedlings were planted at both sites. The seedlings were supplied by OZ Semenoles Liptovský Hrádok (LESY SR, state forest). The mean height of the planted seedlings was about 36 cm, and the mean root collar diameter at 2 cm above ground level was about 6 mm. All seedlings were bare-root plants and were categorised with regard to size and quality before planting.

Seedlings were planted by a contractor (an external crew) to provide a commercial planting (including its advantages and disadvantages). At both sites, seedlings were planted in holes that were about 40 × 40 cm on the surface and dug 15 cm deep using hoe. Seedlings were planted in rows; the in-row and between-row spacing was 0.5 m at the nursery site (where space was limited) and 1.0 m at the clear-cut site. At each site, 600 seedlings were planted in 10 blocks (i.e., 60 seedlings per block). At the nursery site, blocks were placed next to each other. At the clear-cut site, the blocks were uniformly distributed over an area of 3 ha with 20–30 m between blocks.

Seedlings at the nursery site were irrigated during heatwaves and were fertilized with Konifert Extra produced by Agrofert in an amount of 200 g/m^2^ and weeded once each year. The weeding was done manually. Seedlings at the clear-cut site were weeded once each year but were not irrigated or fertilised.

Individual rows (10 seedlings per row) in blocks were treated with one of six treatments (Fig. 1a–c, Table 1); the treatments were randomized among the rows in each block.

**Table 1:** Description of treatments.

Treatment	Commercial name (composition)	Usage	Producer/distributor
Control	No treatment applied to the seedlings		
Chemical	Vaztak active (50 g/l alpha-cypermethrin)	Suspension with a 1% concentration was applied in an approximately spray volume of 60 l/ha (1.5–2.0 l of suspension per 100 seedlings). Seedlings were sprayed with a knapsack sprayer (referred to as top-up spraying) 3 times per year (April, June, August) and 9 times during the entire study	BASF SE Ludwigshafen Germany
Collar	Hylopro anti-weevil protective collar (bioplastic collar)	Biodegradable plastic collar applied in spring 2018. Each collar was opened, placed around the plant base, and then pushed approximately 1 cm into the soil until the lock engaged (Fig. 1a) Locks were checked once each year, at which time open collars were closed	Grube KG Bispingen Germany
Glue	Vermifix (42% polyolefins, 420 g per 1 kg)	One bottle of glue (400 ml) was applied per 70–100 seedlings. Seedlings were treated from the ground to 20 cm height 3 times per year (April, June, August) and 9 times during the entire study (Fig. 1b)	Papírna Moudrý s.r.o Židlochovice Czech Republic
C wax F wax	Eco-wax (Paraffin and additives)	Physical protection by wax coating. Before they were planted at the two sites, seedlings were treated with melted wax (approximately 80 °C; about 5–10 g of wax per seedling that coated the stem from soil level to 20 cm above soil level) by the double fountain machine Heco-V-450NW (ZetaEcotech, Italy)^35^. After wax was applied, the treated stems were immediately cooled with water (Fig. 1c). C wax is standard; the newly developed F wax is more flexible	Norsk wax AS Larvik Norway

### Assessment of damage and condition of seedlings

Seedlings were assessed every year in October, i.e., at the end of the growing season after pest activity had decreased. Damage to seedlings (feeding scars) caused by *H. abietis* adults was evaluated only at the clear-cut site (as noted earlier, *H. abietis* was absent from the nursery site). If adult *Hylastes* spp. bark beetles were detected on roots or stems (this occurred only at the clear-cut site), damaged areas < 50 mm^2^ were attributed to *Hylastes* spp. If the area of stem damage was ≥ 50 mm^2^, the damage was attributed to *H. abietis* even if *Hylastes* spp. beetles were present. The area of damage (mm^2^) on seedlings caused by *H. abietis* was determined using transparent millimetre paper. Only new damage that occurred during the growing season was measured.

Mortality of seedlings was evaluated at both sites. At the clear-cut site, seedlings were assessed as alive, dead (because of *Hylobius*), dead (because of *Hylastes*), or dead (because of other unknown causes). At the nursery site, the seedlings were assessed as alive, dead (because of human activity), or dead (because of other unknown causes).

### Assessment of the condition and quality of wax coating

The state and quality of wax on seedlings treated with C wax and F wax was assessed at both sites at the end of each growing season. For this assessment, we used our own scale (Table 2)^38^.

**Table 2:** Wax condition scale.

Scale value (rank)	Condition of wax
1	Excellent (undamaged wax)
2	Good (cracks in the wax or other damage, but still protecting the seedling)
3	Average (cracks in the wax or other damage, wax has fallen off ≤ 50% of the seedling circumference)
4	Poor (wax has fallen off > 50% of the seedling circumference and does not protect the seedling)
5	Wax missing from the entire circumference of the seedling

### Economic analyses

We also evaluated treatment costs in individual years and total costs (including labour costs). Costs are given in EUR/ha (excluding tax). Individual costs per ha were obtained from three independent sources from the LESY SR state forest and two from private forest sector (Milan Krajči, pers. comm.), and the means of these data were used in the calculations. Costs are based on treating 3000 spruce seedlings/ha.

### Statistical analyses

The data were analysed using R 4.0.3 software and RStudio version 1.3.1093. Plots were made using the ggplot2 package^55^. Data were processed using the dplyr R package version 1.0.2.^56^. All mixed-effect models were calculated using lmer and glmer functions of the lme4 package, version 1.1-19^57^.

#### Damage and mortality of seedlings

Damage and mortality caused by *H. abietis* adults were evaluated only at the clear-cut site. To study the effect of treated (chemical, collar, glue, C wax, and F wax) and control seedlings on *H. abietis* feeding damage and mortality, we excluded dead undamaged seedlings and seedlings that died due to *Hylastes* or “other” factors.

Damages and mortality were measured from multiple blocks: explanatory variable—block (10 levels). In the period 2018–2020: explanatory variable—year (3 levels).

##### Seedling damages

At the clear-cut site, the effect of treatments on damage by *H. abietis* feeding was analysed using a linear mixed model (LMM), and the parameters were estimated using the residual maximum likelihood method (REML). The response variable, i.e., feeding damage (mm^2^), was transformed using the Box–Cox transformation (R, package bestNormalize; version 3.4.1)^58^ to improve the homogeneity of the variance and the normality of the distribution. Seedlings treated with the chemical suspension were excluded from the analyses because the damage ranged from small to none, with a median value of zero throughout the entire experiment.

We first developed a series of alternative mixed effect models that included different combinations of explanatory variables. Models were compared using the Akaike information criterion (AIC) and ANOVA. Random effects variance components were estimated using REML. Models with various fixed effects were compared using a maximum likelihood estimation (ML).

Random effect: We fitted the maximal complexity of the random effect structure. We tested the hypothesis that the strength of the treatment's effect (slope) on the response variable varies between blocks and years. The least significant terms were dropped. Then the variables block and year were included as crossed random effects (intercept).

Fixed effect: Variable’s treatment and year significantly improved model parameters and were included in the selected model.

Finally, the selected model's variables, treatment, and year were fitted as fixed effects and block as crossed random intercept. The selected model shown in R notation format:

$$\sim {\text{treatment }} + {\text{ year }} + \, \left( {{1}|{\text{block}}} \right).$$

##### Seedlings mortality

The effects of treatments on seedling mortality were analysed using the generalized linear mixed model (GLMM) fitted using the maximum likelihood Laplace approximation with a binomial distribution and a logit link function. Seedling mortality (alive/dead) was the response variable in the model. Treatment and the study periods were used as a fixed effect, and blocks were used as a random effect.

Treatments were compared using the Tukey method for comparing family estimates. Pairwise differences of least squares means were computed using the emmeans R package (version 1.5.4.)^59^.

Statistical assumptions of the selected models were simulated and graphically validated using DHARMa package for residual diagnostics of hierarchical regression models. All the assumption tests provided by the package were insignificant^60^.

#### Assessment of wax condition and quality

The number of seedlings differed between the years of the experiment. Dead seedlings were excluded from the analyses because they did not grow. Wax quality was compared between wax treatments (C wax and F wax) using the Mann–Whitney U test with the p-value estimated by normal approximation with a continuity correction.

To test how the wax quality deteriorated during the experiment, we used the Wilcoxon–Pratt signed-rank p test with an exact type I error estimate, using the asht R package^61^. The test assumes that differences between paired samples should be distributed symmetrically around the median. We calculated the differences and plotted them in histograms. All differences were distributed approximately symmetrically.

## Results

### Effectiveness of treatments at the clear-cut site

**Figure 2:**
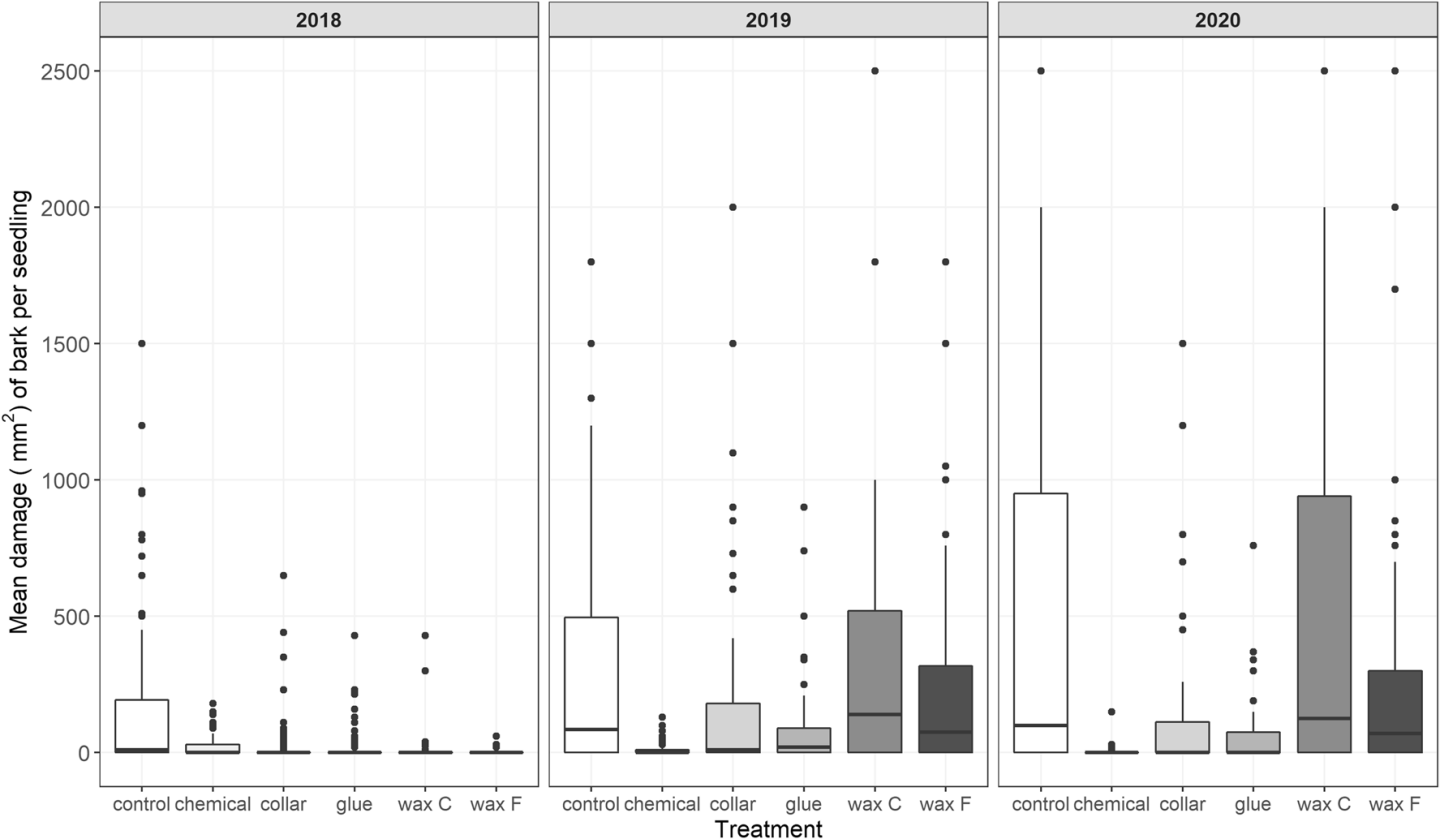
Effect of treatments on the area damaged per seedling by *H. abietis* at the clear-cut site.

In 2018, the mean damage per seedling across all treatments was relatively low (Fig. 2). Damage was highest for control seedlings and lowest for seedlings treated with F wax and C wax. In 2019, the damage across all treatments except the chemical treatment substantially increased. Seedlings treated with C wax and control seedlings were the most damaged in 2019. In 2020, the mean damage per seedling across all treatments (excluding the chemical treatment) was the highest in the 3 years. Seedlings treated with C wax and control seedlings were again the most damaged. The lowest levels of damage were recorded on seedlings treated with the chemical and the glue.

Based on the area of feeding scars, the chemical treatment provided the best protection against *H. abietis* damage (Table 3). For this treatment, we recorded almost no damage, i.e., the median damage was equal to zero during the whole experimental period. Only a few seedlings were attacked. Because we analysed only damaged seedlings, data from the chemical treatment were not included in the LMM.

**Table 3:** Areas of feeding scars by *Hylobius abietis* on seedlings treated with treatments.

Treatment	2018		2019		2020	
	Mean	SD	Mean	SD	Mean	SD
Control	149.1	275.1	336.2	450.4	480.7	708.1
Chemical	21.3	38.3	13.8	30.0	5.1	20.0
Collar	27.2	89.2	211.8	409.1	134.8	296.8
Glue	21.4	63.1	81.6	164.2	63.9	124.6
Wax C	8.7	52.4	338.2	497.1	501.4	669.7
Wax F	1.1	6.9	232.7	359.8	260.9	473.2

Treatment (p < 0.001, χ^2^ (5) = 49.28) had a significant effect on the area of feeding scars caused by *H. abietis.* The average damages (p < 0.001, χ^2^ (2) = 54.49) were higher each following year of study. Only glue-treated seedlings and those protected with collars had significantly less damage than control seedlings (Table 4). The glue treatment performed significantly better than either of the wax treatments and also significantly better than the collar treatment (Table 5). The difference between the glue and collar treatment was lower than the differences between the glue and the wax treatments. Damage was significantly lower with the collar treatment than with either of the wax treatments.

**Table 4:** Results of mixed linear models (LMMs) relating seedling damage to five treatments (excluding the chemical treatment) at the clear-cut site.

Treatment	Estimate	95% CI	SE	p
Control (Intercept)	− 0.321	− 0.58 to − 0.06	0.133	0.023
Collar	− 0.425	− 0.64 to − 0.17	0.135	0.006
Glue	− 0.807	− 1.06 to − 0.55	0.132	< 0.001
F wax	− 0.182	− 0.44 to 0.12	0.133	0.282
C wax	− 0.030	− 0.29 to 0.33	0.137	0.673
year 2019	0.589	0.37 to 0.81	0.11	< 0.001
year 2020	0.903	0.66 to 1.14	0.12	< 0.001

**Table 5:** Comparison of damage caused by pine weevil between treatments at the clear-cut site; the Tukey method was used to compare family estimates.

Contrast	Estimate	SE	95% CI	p
Control–Collar	0.373	0.136	0.020 to 0.724	0.0097
Control–Glue	0.800	0.133	0.456 to 1.143	< 0.0001
Control–C wax	0.143	0.135	− 0.204 to 0.491	0.6420
Control–F wax	− 0.058	0.138	− 0.415 to 0.300	0.9994
Collar–Glue	0.427	0.146	0.049 to 0.804	0.0562
Collar–F wax	− 0.229	0.144	− 0.603 to 0.144	0.4158
Collar–C wax	− 0.431	0.148	− 0.81 to 0.048	0.0441
Glue–F wax	− 0.656	0.142	− 1.024 to − 0.288	0.0001
Glue–C wax	− 0.858	0.145	− 1.233 to 0.481	< 0.0001
F wax–C wax	− 0.201	0.14	− 0.563 to 0.161	0.8062

Seedlings protected with C or F wax had lowest damage in the first year of the experiment. Protection by the wax coatings, however, lasted only 1 year. In the second and third years of the experiment, C wax had no protective effect, and C wax-treated seedlings were damaged to the same extent as control seedlings.

### Seedling mortality as affected by the treatments

#### Mortality at the clear-cut site

**Figure 3:**
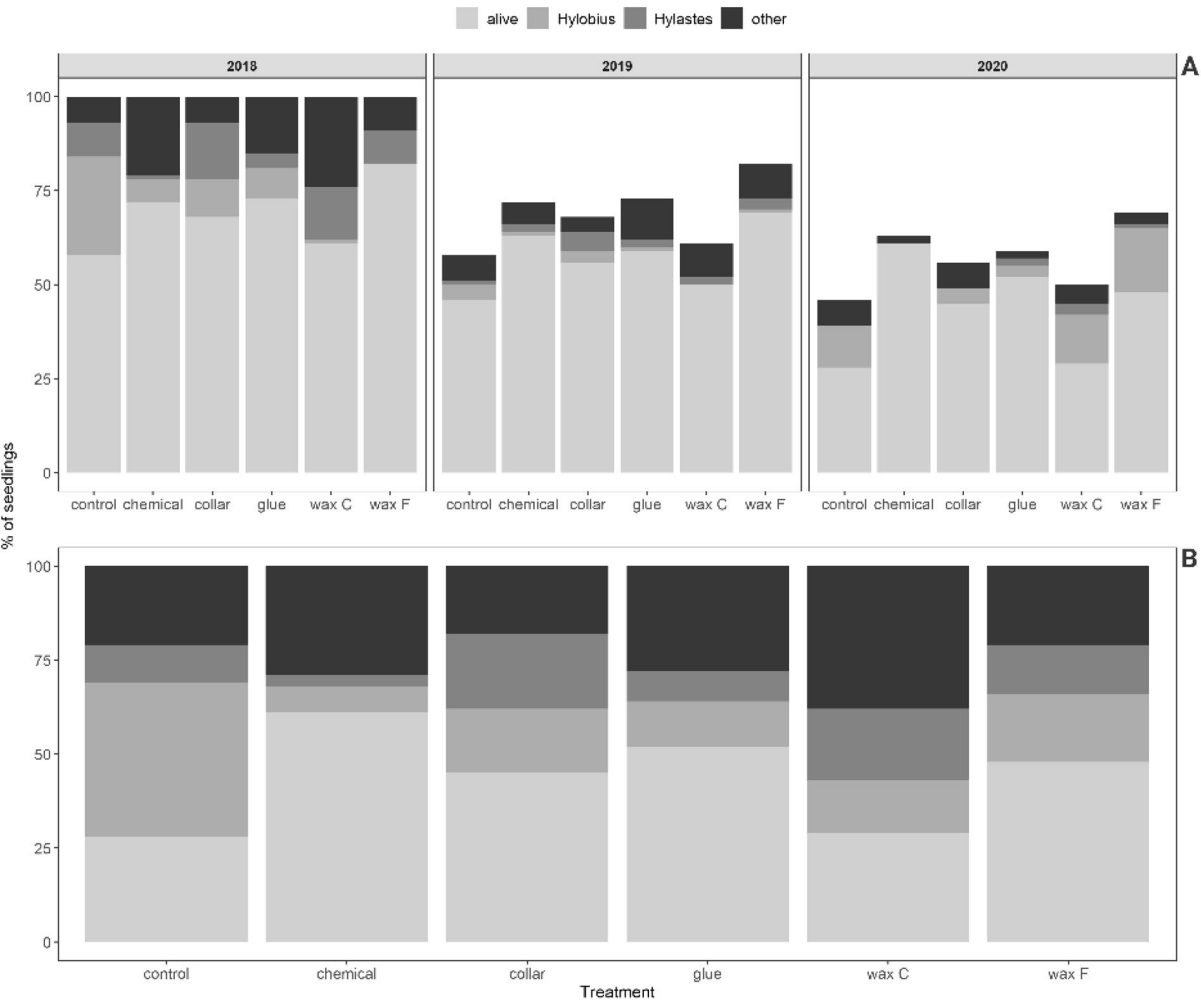
(**A**) Number of seedlings that remained alive or died (for the indicated reasons) as affected by treatments at the clear-cut site in each of the 3 years of the experiment. (**B**) Total seedling mortality over the 3 years of the experiment as affected by treatments at the clear-cut site.

In 2018 at the clear-cut site, the mortality across all treatments including the chemical treatment was 31% (Fig. 3A). Mortality was highest with the control treatment followed by the C wax treatment, and was lowest with the F wax treatment. In 2019, the mortality across all treatments was 17%. Mortality in 2019 was lowest with the chemical treatment (Fig. 3A). In 2020, mortality across all treatments was 23% (Fig. 3A). Mortality in 2020 was highest with the C wax and control treatments and was lowest with the chemical treatment (Fig. 3 A).

In 2018, the highest number of seedlings died because of *H. abietis* damage to control seedlings. In 2019, *H. abietis* damage increased (Fig. 2), and resulted in high levels of mortality to control seedlings; *H. abietis* damage also caused high levels of mortality of control seedlings and seedlings treated with either of the waxes in 2020 (Fig. 3A). Damage caused by *Hylastes* occurred in all years and with all treatments, and was lowest with the chemical treatment (Fig. 3A,B).

Mortality of seedlings at the end of the 3-year experiment significantly differed among the treatments (p < 0.001, χ^2^(5) = 31.15) and years (p < 0.001, χ^2^(2) = 24.21) at the clear-cut site. Mortality was generally lower for seedlings treated with the chemical, waxes, or collar than for the control seedlings (Table 6). The highest odds ratio of survival was found for the seedlings treated with chemicals (Table 6). There was no difference in the mortality of control seedlings and those treated with C wax. Treatments with glue and F wax significantly improved the survival odds. Seedlings with collars had a higher odds ratio than control seedlings. The effect appears to be strong, but because the lower limit of the confidence interval is below 1.0, the overall effect is not that significant (Table 7).

**Table 6:** Estimated parameters for the effect of treatments on the mortality of seedlings at the clear-cut site.

Treatment	Odds-Ratio	95% CI	SE	p
Control (intercept)	0.73	0.31–0.89	0.146	0.140
Chemical	0.345	0.22–0.55	0.08	< 0.001
Glue	0.458	0.30–0.71	0.10	< 0.001
Collar	0.588	0.38–0.91	0.13	0.016
C wax	0.866	0.57–1.32	0.10	0.512
F wax	0.454	0.29–0.70	0.19	< 0.001
year 2019	0.49	0.36–0.68	0.08	< 0.001
year 2020	0.75	0.55–1.14	0.12	0.068

**Table 7:** Comparison of mortality between treatments at the clear-cut site based on the Tukey method for comparing family estimates. The log odds were exponentiated to obtain the odds-ratios (OR).

Contrast	Odds-Ratio	CI	SE	p
Control–Chemical	2.895	1.48–5.65	0.6793	0.0001
Control–Glue	2.179	1.14–4.13	0.4895	0.0069
Control–Collar	1.698	0.90–3.17	0.3731	0.1526
Control–F wax	2.202	1.17–4.12	0.4855	0.0046
Control–C wax	1.153	0.62–2.11	0.2461	0.9853
Chemical–Glue	0.753	0.37–1.50	0.1828	0.8511
Chemical–Collar	0.586	0.29–1.15	0.1402	0.2227
Chemical–F wax	0.761	0.38–1.50	0.1815	0.8618
Chemical–C wax	0.398	0.20–0.77	0.0927	0.0011
Glue–Collar	0.779	0.40–1.49	0.1787	0.8865
Collar–F wax	1.011	0.52–1.94	0.2315	1
Glue–C wax	0.529	0.28–0.99	0.1179	0.0491
Collar–F wax	1.297	0.683–2.46	0.292	0.8582
Collar–C wax	0.679	0.36–1.26	0.1482	0.4838
F wax–C wax	0.524	0.28–0.97	0.1143	0.0359

#### Mortality at the nursery site

**Figure 4:**
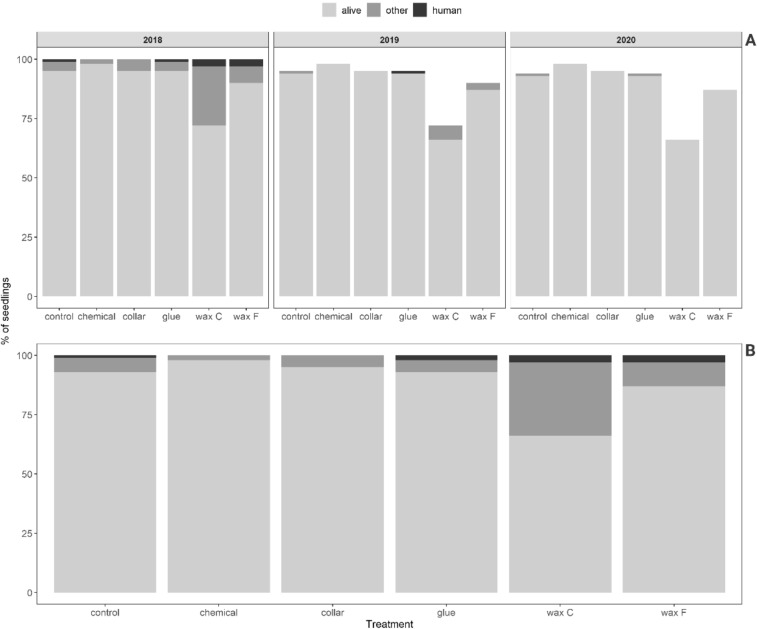
(**A**) Mortality of seedlings at the nursery site in each of 3 years of the experiment as affected by the treatments. (**B**) Total seedling mortality at the nursery site over the 3 years of the experiment as affected by the treatments.

Mortality was lower at the nursery site than at the clear-cut site because of the absence of *H. abietis* damage or *Hylastes* damage at the nursery site (Fig. 4A,B). In 2018 at the nursery site, 47 seedlings died due to unknown (other) reasons, and eight died due to accidental damage during handling. One-fourth of the seedlings treated with C wax died in the first year. In 2019, only 10 seedlings (mostly coated with C or F wax) died for unknown reasons, and one seedling died due to handling. In the last year of the experiment (2020), seedlings were well established, and only 2 died (for unknown reasons; Fig. 4A,B).

### Wax condition and quality as affected by wax type and year

#### Effect of wax type on wax quality

At the clear-cut site, there were no significant differences in wax quality between C and F wax in any year of the experiment (2018, W = 2680, p-value = 0.423; 2019, W = 1935, p-value = 0.236; 2020, W = 673.5, p-value = 0.800).

At the nursery site, wax quality significantly differed between C and F wax in 2019 and 2020; quality was higher for F wax than for C wax in both years (2018, W = 3231.5, p-value = 0.8429; 2019, W = 3401, p-value = 0.034; 2020, W = 3505, p-value = 0.01).

#### Effect of year on wax quality

**Figure 5:**
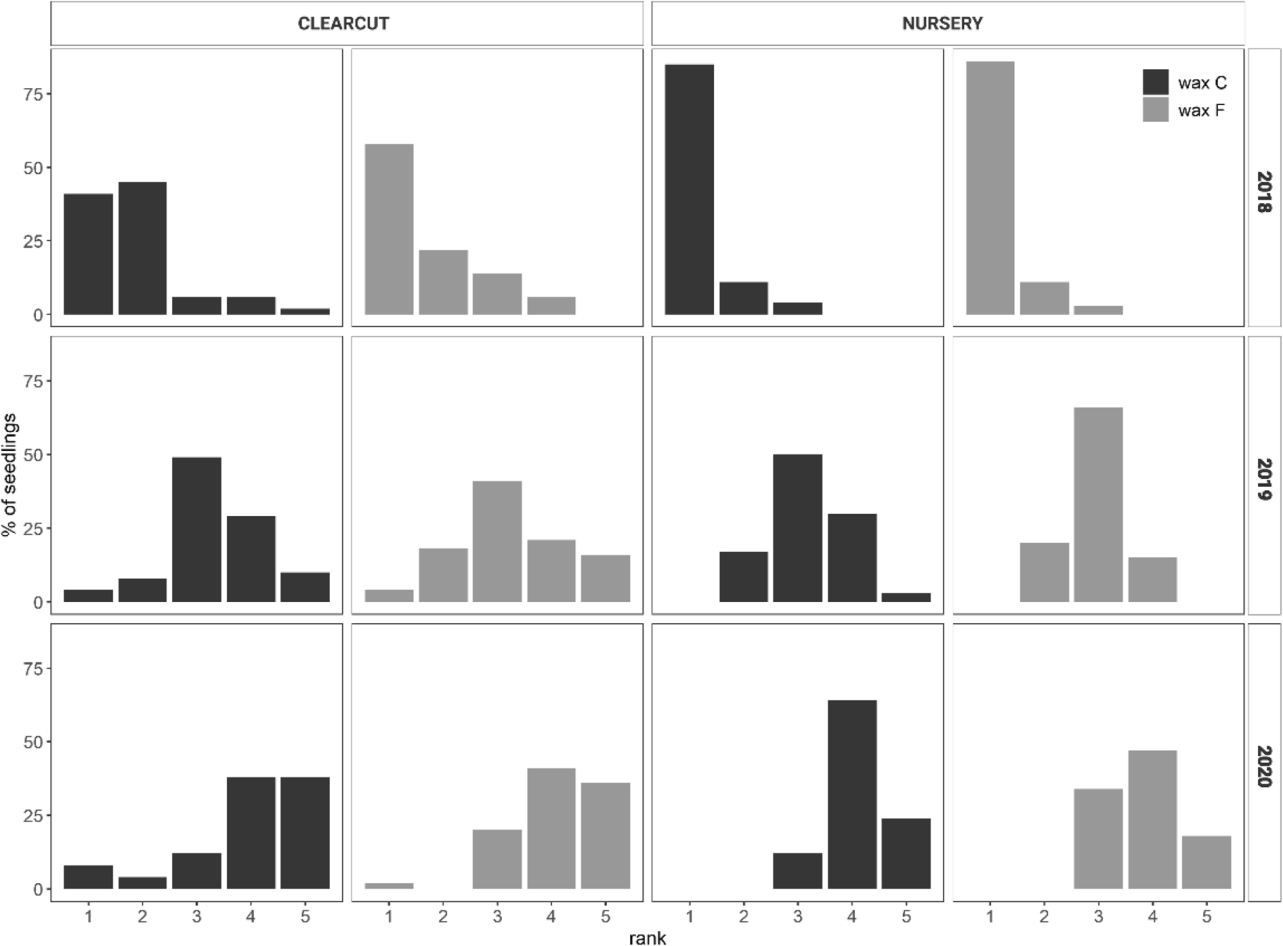
Distribution of quality (ranked on a scale from 1 to 5 for the two wax types at two sites and for 3 years of the experiment. Wax quality was ranked on a scale from 1 highest quality to 5 lowest quality).

The distribution of wax quality significantly differed among the years of the study for both types of wax and for both sites. At the clear-cut site, the quality of C wax decreased with time (Fig. 5), i.e., wax quality was higher in 2018 than in 2019 (n = 47, Z = − 5.8576, p < 0.001) and was higher in 2019 than in 2020 (n = 24, Z = − 4.2666, p < 0.001). The same reductions were evident for F wax, i.e., wax quality was higher in 2018 than in 2019 (n = 68, Z = − 6.8978, p < 0.001) and was higher in 2019 than in 2020 (n = 44, Z = − 5.2382, p < 0.001).

The results from the nursery site were similar to those from the clear-cut site. The quality of C and F wax significantly decreased with time. For C wax: 2018/2019 (n = 66, Z = − 7.2349, p < 0.001; 2019/2020 (n = 66, Z = − 4.1242, p < 0.001). For F wax: 2018/2019 (n = 87, Z = − 8.3528, p < 0.001); 2019/2020 (n = 87, Z = − 7.4788, p < 0.001). In the first year, quality in the best rank 1 was higher for F wax than for C wax (Fig. 5).

### Economic evaluation of the treatments

**Figure 6:**
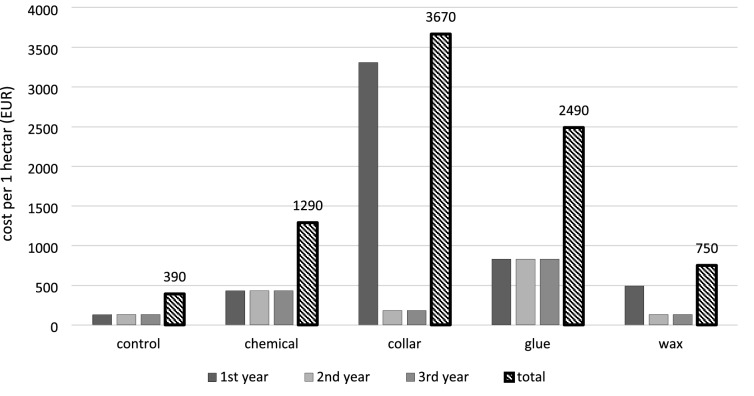
Costs per ha to protect spruce seedlings with the treatments. Treatment costs per ha are for the standard number of 3000 seedlings/ha. All columns include standard costs for weeding once each year (130 EUR/ha). Price for 1 l of Vaztak Active is approximately 32 EUR (required amount = 0.6 l/ha). Discount price for 1 bottle of Vermifix spray is approximately 2.3 EUR (required amount = 45 bootles/ha). Price for 1 collar is approximately 1 EUR (required amount = 3000 collars/ha). Costs per 1 waxed seedling are 0.12 EUR (material and service). The presented prices are valid for Slovakia (year 2021).

All costs needed for the application of the treatments are indicated in Fig. 6. Weeding once each year costs 130 EUR/ha (in total 390 EUR/ha for 3 years), and these costs were added to all treatment types (including the control).

The costs were lowest for the wax treatments (750 EUR/ha for 3 years). Wax coating was applied in the first year (360 EUR/ha), and was not applied again because this treatment cannot be repeated in the field.

Chemical treatment of seedlings was relatively inexpensive (1290 EUR/ha for 3 years), even though it was applied three times each year. A single chemical treatment (material and labour) costs 100 EUR/ha.

Treatment of seedlings with glue was relatively expensive (2490 EUR/ha for 3 years). According to our calculations, a single treatment costs approximately 233 EUR/ha (material and labour).

Mechanical protection of seedlings with collars was the most expensive (3670 EUR/ha for 3 years). These biodegradable collars are very expensive (ca. 1 EUR each), and they must be checked at least once each year (approximately 50 EUR).

## Discussion

Most recent experiments that compared chemical and non-chemical treatments for protection of seedlings against *H. abietis* have been conducted in the UK^11,24,26,32,33^ and Slovakia^19,21,38^. A comprehensive comparison of efficacy based on our results and our experience with all available and possible protection methods in Central Europe is provided in the following sections.

### Chemical treatment

The application of pesticides in forestry will be gradually restricted or forbidden (different certification schemes such as Forest Stewardship Council (FSC) and Programme for the Endorsement of Forest Certification (PEFC); European Commission policies described in Refs.^11,19,24,26^ even though much less pesticide is applied to forests than to agricultural crops^26,62^. Some effective insecticides (cypermethrin, alpha-cypermethrin) have been replaced by less toxic insecticides^11,26,33^ and by ULV (ultra-low volume) application techniques^24^. Using of insecticides is restricted in national parks^19^ and is restricted to treat trees only in nurseries before they are planted in the forest^26^. In our study, we used a non-systemic pyrethroid insecticide, alpha-cypermethrin. Despite its disadvantages (described above and in Table 8), alpha-cypermethrin provided the best protection against *H. abietis* (Figs. 2, 3A,B). In the third year, alpha-cypermethrin application resulted in almost zero damage by *H. abietis* to seedlings (Fig. 2), and the lowest mortality of seedlings at both sites (Fig. 4). The high effectiveness of insecticides has been described by the authors cited above, which indicates that a similarly effective alternative may be difficult to find. Moreover, chemically treated seedlings were the least damaged by *Hylastes* spp., which attack the roots (Figs. 3, 4). We suspect that the alpha-cypermethrin provided protection against *Hylastes* spp. by running down the stem to the root neck.

**Table 8:** Strengths and weaknesses of the treatments used to reduce *H. abietis* damage to seedlings in this study.

Treatment mark	Strengths	Weaknesses
Chemical	Cheap and effective (partly effective also against *Hylastes* spp.) Simple and fast application Different forms of application (pre-planting, post-planting) Treatment can be repeated in the field	Environmental impact Staff safety Chemicals are restricted and will be probably banned in forestry in some countries (see cited works in Introduction) Need to repeat treatments (2–3 times per season)
Glue	Good effectiveness Simple application New method	Costs per ha Potential phytotoxicity Need for more tests
Collar	Low environmental impact Easy to set up	Costs per ha Some collars do not remain locked, checking once a year is necessary If the pine weevil gets into the collar, plant damage can be substantial Biodegradability is debatable
Wax type C and F	Low environmental impact Costs per ha (only one treatment) Excellent effectiveness in the first year F composition seems better More layers can be put on	Not suitable at sites with high population densities Quality of treatment—staff needs to be careful during the whole process from waxing up to planting Phytotoxicity in the case of insufficient cooling Wax cracking and falling off in the second season Treatment can not be repeated in the field

### Wax treatment

In the first year of the study, wax provided the best protection among all of the treatments; in that year, seedlings treated with either wax type had the lowest mortality caused by *H. abietis* and the smallest area of feeding scars (Fig. 2). In the second year, the wax began to crack and fall off (Fig. 5). In the third year, the wax provided no protection, and damage for wax-treated seedlings was similar to damage for untreated (control) seedlings (Fig. 2). Although no significant difference was found between the damage of seedlings treated with different wax types against *H. abietis* (Table 5), seedling mortality was lower for F wax than for C wax (Table 7), probably because of the better elasticity of F wax (J.M. Petersson, pers. comm.). This difference in the efficacy of the two wax types was confirmed at the nursery site in 2019 and 2020, where significant differences in quality were found between F and C wax types. At the clear-cut site, however, the two types of waxes did not differ in quality. Moreover, the mortality of seedlings was lower for F wax than C wax at both sites (Figs. 3, 4), probably because of higher phytotoxicity (as indicated by mortality caused by “other, unknown factors”) with C wax. In contrast, Eriksson^27^ found that seedlings treated with the F type had a significantly higher rate of damage caused by “other, unknown factors” than seedlings treated with the C type. Apart from our work, Eriksson^27^ has been the only study that tested the F type of wax. Many studies have compared wax with other treatments and have found that wax had promising effectiveness against *H. abietis*^19,27,30,38,42,62–65^, including our studies. This was confirmed in the first year of our study, but the quality and hence the protective characteristics of wax rapidly decreased in subsequent years. Most of the cited works monitored wax effectiveness only for 1 or 2 years. *H. abietis* imagoes, however, may remain at the site for up to 4 years^8^, which can be a problem for seedlings treated with wax, especially in the third year (as in our case); when the wax falls off (Fig. 5), the seedlings are no longer protected (Fig. 2). This is the greatest weakness of wax treatments (Table 8). Problems with cracks and falling off have been reported by forest managers from Slovakia, the Czech Republic, and Austria (pers. obs.). Similar results have been presented^26,32^ who found that wax quality declined in the cold weather of the UK. This is most probably the reason for wax cracking in our study (Fig. 5), because the chosen sites (nursery and clear-cut) are located in areas with harsh mountainous conditions (above 800 and 1000 masl, respectively).

The producer suggests that the ideal thickness of the wax layer is 1.5 mm (J. M. Peterssen, pers. comm.), because this thickness resulted in less cracking than thinner layers^66^. The thickness of the wax layer in this study was consistent with the producer’s recommendations.

Wax temperature during the application is approximately 80 °C, and treated seedlings must be quickly cooled with water to avoid damage to the seedlings^36^. During this procedure, mistakes can occur, which could have caused increased phytotoxicity and mortality at both sites in the first year of our study (Figs. 3A, 4A). We observed that the staff handling the seedlings damaged them during waxing, transport, storage and planting (Table 7). It is necessary to increase staff awareness of the need for careful handling of seedlings during and after waxing. In other experiments, we frequently observed that the wax was damaged immediately after planting. Another frequent mistake is that the seedlings are planted too deep, and only few centimetres of the wax layer remain above the ground. These mistakes may be reduced by staff training, which should include the reading and reviewing of a manual/leaflet that explains potential problems. Wax cracking can be partially reduced by applying additional layers (two layers crack less than one layer) (G. Nordlander, pers. comm.). A further detailed comparison of wax and insecticides based on reports^27,30,42,62–64^ is discussed in our previous study^19^.

Willoughby^26^ found that wax provided variable protection against *H. abietis* and that protection was poor at sites with abundant *H. abietis* populations. At sites with low to intermediate populations of *H. abietis*, however, protection was sufficiently promising to warrant further experiments with wax^26,32^. The cracking of the wax layer and the high population of *H. abietis* at the clear-cut site^54^ could explain the weak protection provided by wax in the current study.

### Glue treatment

Vermifix glue has been studied only by the authors of the current study^19,38^. During the first experiments in 2015 and 2016 (unpubl. data), the glue caused relatively high phytotoxicity on spruce seedlings. The producer subsequently managed to reduce this problem because, in the current study, seedling mortality was not higher with glue than with other treatments at either site even though multiple coatings of glue were applied (3 times every year, 9 layers in total) (Figs. 3, 4). Moreover, glue significantly reduced *H. abietis* feeding damage, and provided the second-best protection (Fig. 2). To date, only a few other studies have evaluated the effects of glue treatment of seedlings on *H. abietis* feeding damage. Eriksson^27,62^ found that glue (Bayer) prevented adults from feeding on seedlings. Similar feeding barriers, which are applied by coating, and which were tested against *H. abietis*, include Polymer^24^, Flexcoat^26,34^, Södra^27^, and Conniflex; the latter material is widely used in Sweden^3,31^.

### Collars

Other feeding barriers include collars. The type of the collar used in our study (Table 1, Fig. 1b) was tested under Central European conditions for the first time. We found that the collars protected seedlings better than wax (Fig. 2). The producer states that the type of plastic used to construct the collars is biodegradable and will last in the field for 4 years (Table 1), but our previous experiments (unpubl. data) indicate that these collars do not degrade even after 4–5 years (pers. obs.). The deploying of these collars was also problematic because the locks did not remain closed (the collars were checked once each year). In some cases, *H. abietis* adults climb over the collar and cause substantial damage before the damage is detected. Similar findings were reported from the UK^24^. The major disadvantage of collars, however, is their price (Table 8, Fig. 6), and we therefore do not recommend their use.

A detailed comparison of the effectiveness of different types of feeding barriers, which have been developed mainly in Sweden^29^, has been recently presented^21^ and is therefore not presented here because of space limitations. In general, these barriers are relatively expensive, must be checked frequently, and are not suitable in the areas with high population densities of *H. abietis*. The barriers warrant further research, however, as non-chemical alternatives for control of *H. abietis*.

### Practical and economic comparison of the treatments

Based on our previous experiments (cited in this work and unpubl. data) and the current results, we have summarized the weaknesses and strengths of the applied treatments in Table 8. Multiple comparisons with other treatments are presented in new studies^24,26,33^.

We found that application is substantially cheaper for wax and insecticides than for glue and collars (Fig. 6). In our case, the costs per one waxed seedling was 0.12 EUR using the fountain machine, in addition the producer states that the cost is 0.05 EUR (or 0.05–0.09 EUR per tree^39^ using the new wax machine (semi-automatised link)^66^ with costs per ha equal to 110 EUR.

In our study, we applied the insecticide three times per year, and the costs were still low. Our findings also support Hardy^24^, who found that the tested insecticides were cheap and effective. The collar and glue used in our study are expensive (Fig. 6) (3670 and 2490 EUR per ha, respectively). The total average costs of planting are 1744 EUR per ha in Slovakia (State Forests, pers. comm.). Considering this information and the costs for collar or glue treatments, it may be cheaper to replant an area than to use collars or glue.

The initial costs of seedling protection can be reduced by delaying restocking until *H. abietis* population levels have fallen^32^, but this approach generates costs for weeding, increases costs for planting due to worsened conditions, and delays wood production for future generations. Costs could also be reduced using a management support system, which helps foresters predict and reduce damage; such a system has been developed by Moore^67^ for UK conditions. Another detailed comparison of different treatments can be found in Willoughby et al.^11^.

### Recommendations for forest managers

Based on our experience and considering the pressure to reduce insecticide applications in forests, we recommend the following for forest management in Central Europe:

- In spite of their ecological disadvantages, insecticide application will for the near future be the least expensive and most effective way to protect seedlings against *H. abietis* in areas with high population densities.
- The negative effects of insecticides on the forest environment can be reduced by treating seedlings with the insecticides before they are planted in the field (e.g., by dipping them in insecticides in the nursery). Depending on the insecticide, such a treatment remains effective for 5 months^68^, or for 3–7 months^11,34^. Viiri^69^ also found that dipping in an insecticide was superior to spraying an insecticide for controlling *H. abietis*. According to our experience and those of researchers^11,26^, spraying is effective for 1–3 months.
- Environmental impacts of insecticides could be reduced by applying less toxic insecticides^11,26^ or by using ULV application methods^24^.
- In areas with low to moderate *H. abietis* damage, different types of feeding barriers can be used including new types of feeding barriers that are comparable in cost to wax and insecticide treatments.
- Wax is an inexpensive and a good alternative to insecticides, but its negatives need to be eliminated (wax cracks, falls off, and can be phytotoxic). Wax is not suitable for sites with extremely high *H. abietis* population densities. Wax may also be unsuitable for areas with harsh conditions^26^.
- Vermifix glue is the most effective alternative to chemical treatments. Its potential to protect spruce seedlings has been confirmed^19,38^, but although its spray application is effective, it is relatively expensive. Theoretically, the glue can be applied in a less expensive manner, or eventually can be mixed with sand to provide an abrasive repellent against *H. abietis* feeding. Further research on glue is warranted.
- A combined use of wax in the first year of planting (according to most authors, wax is effective as long as it remains in place) and of insecticides (if allowed) or glue in the second (third) year should be considered. This combination would be cheaper and less harmful to the environment than the repeated application of insecticide. Another possibility is delayed restocking with waxed seedlings.
- Research on bio-pesticides and new methods of their application (e.g., using the bio-insecticide carrier described in Lalík^70^ should be supported).
- Maximum use of silvicultural practices that reduce feeding damage by *H. abietis* on planted seedlings. These practices include natural regeneration, weed removal, delayed restocking, planting mixed forests, scarification, and others that are described in the works cited in the Introduction or in the review^21^.

## Conclusions

In the current study, we found that insecticide application was the most effective and least expensive way to protect seedlings against *H. abietis*, but the use of insecticides in European forestry will be severely restricted or even banned in the future. The glue spray used in our study showed promising results for seedling protection and can be easily applied.

Collars provided moderate protection, but they are expensive, their locks must be checked annually, and the biodegradability of the collars used is questionable. We therefore do not recommend using this type of collar in its present form.

The major weakness of wax is its potential to crack and fall off the seedling in the second half of the second season after waxing. Despite the promising results, wax effectiveness in this study was not sufficient in the second and the third year. The newer F type achieved better results than the standard C type, and we therefore suspect that further research could improve wax performance; this research could include the application of two wax layers, the development of a more flexible wax, the renewal of the wax layer in the field, and the reduction of wax phytotoxicity.

We also see some promise for combining the tested methods, e.g., wax could be used to protect seedlings in the first year, and the spraying of glue or insecticides (if allowed) could be used to protect seedlings in the second year. Although the current study and the papers cited herein have provided many answers, many questions about how to protect coniferous seedlings against *H. abietis* remain unanswered. Additional research is clearly needed to improve some of the existing protection methods and to develop new protection methods.
